# Experiment for Measuring Mechanical Properties of High-Strength Steel Sheets under Cyclic Loading by V-Shaped Double-Shear-Zone Specimens

**DOI:** 10.3390/ma16134645

**Published:** 2023-06-27

**Authors:** Geng Yan, Yanli Lin, Shuo Wang, Enqi Xu, Zhubin He, Kelin Chen, Shijian Yuan

**Affiliations:** 1 School of Mechanical Engineering, Dalian University of Technology, Dalian 116024, China; 32004268@mail.dlut.edu.cn (G.Y.); wshuo98@163.com (S.W.); xuenqi@mail.dlut.edu.cn (E.X.); hezb@dlut.edu.cn (Z.H.); kchen@dlut.edu.cn (K.C.); shijianyuan@dlut.edu.cn (S.Y.); 2 State Key Laboratory of High-Performance Precision Manufacturing, Dalian 116024, China

**Keywords:** simple shear, large strain, Bauschinger effect, cyclic loading, Q890 high-strength steel

## Abstract

The simple shear test shows significant advantages when measuring the hardening and shear properties of thin sheet metal at large strains. However, previous shear tests had an end effect caused by local stress concentration and a boundary effect caused by deformation overflow, resulting in non-uniform strain distribution in the shear zone. Therefore, a unique V-shaped double-shear-zone specimen is proposed to measure the Bauschinger effect under cyclic shear loading conditions in this paper. Simple shear experiments and three different types of cycle shear experiments are conducted to analyze the uniformity of deformation in the shear zone and the effect of pre-strain and the number of cyclic loads on the Bauschinger effect of Q890 high-strength steel sheets. The results indicate that the proposed V-shaped double-shear-zone specimen can still maintain uniform shear deformation in forward/reverse cyclic loading experiments, even at large strains. Q890 high-strength steel exhibits a significant Bauschinger effect, which is more pronounced with the increase in shear pre-strain and loading cycles. The results of this paper provide a new approach for studying the hardening characteristics under large strain and the mechanical properties under cyclic shear loading for metal sheets.

## 1. Introduction

With the increasing prominence of the energy crisis and awareness of environmental protection, lightweight design has become a critical aspect of Advanced Manufacturing Technology (AMT) [1,2]. To achieve the goal of ensuring performance while reducing weight, advanced high-strength steels (AHSS) with high specific strength have been used to manufacture integrated structural parts, and have been widely applied in manufacturing fields such as the aviation, aerospace and automotive industries [3,4,5]. However, the forming of such components is challenged by two factors: firstly, the higher strength of the material leads to significant springback after forming [6]; secondly, the complex structure requires a complicated forming process, which often requires repeated loading and unloading during the forming process. Therefore, accurately describing the mechanical properties of such materials under stress cycles, i.e., the precise measurement of the Bauschinger effect [7], is of great significance for accurately predicting springback, formulating process routes, designing forming dies and constructing the constitutive models [8,9,10,11].

To reveal the mechanical properties and the Bauschinger effect of sheet metal under cyclic loadings, several different experimental methods have been developed by many researchers. These experimental methods can be mainly classified into three categories: (1) the in-plane tension–compression test; (2) the bending–reverse bending test; and (3) the in-plane cyclic simple shear test.

The in-plane tension–compression test is widely used in the study of the Bauschinger effect [12] because it provides a uniform strain distribution, and the specimen shape is simple and easy to prepare. However, this test method often fails to measure large strains due to the early buckling of the specimen during compression [13]. In order to suppress the out-of-plane buckling, Kuwabara [14] designed a device for placing solid plates on both sides of the specimen’s surface to apply a clamping force. For the thinner sheets, Yoshida [15] used a method of bonding multiple specimens together for tension–compression testing to suppress buckling. However, there is still a gap between the fixture of this kind of experimental device and the chuck of the testing machine, which leads to buckling in the unsupported region during the tensile process [16]. To eliminate unsupported regions and improve the strain measurement range, Cao et al. [17] developed a novel double-wedge fixture with a spring for tension–compression experiments, which allows the specimen with double-side fins to move freely in the loading direction while applying a clamping force across the specimen’s surface. However, the biaxial effect caused by the clamping force of the fixture and the friction between the fixture and the sample affect the accuracy of the test results. In addition, since the uniaxial tensile specimens produce necking, the in-plane tension–compression experiments cannot obtain the mechanical properties of the specimen after necking [18,19].

During the bending–reverse bending test, the inner material of the sheet undergoes compression–tension deformation while the outer material undergoes tension–compression deformation, avoiding buckling of the sheet during compression, and thus the Bauschinger effect under large strain can be obtained. Zhao et al. [20] studied the hardening behavior and Bauschinger effect of high-strength steel under large strain using the three-point bending–reverse bending test. Eggertsen and Mattiasson [21] evaluated the performance of five different hardening laws based on cyclic three-point bending experimental results. Instead of using the cyclic bending test, Zang et al. [22] calibrated the hardening parameters inversely by measuring the three-point bending springback curve of the pre-stretched sheet and combining it with the finite element technique. Compared to the previous work, this method simplifies the test process. However, this test method needs to establish the constitutive model of the material in advance, which is not suitable for studying the mechanical properties of materials whose constitutive models are not available. Additionally, the cyclic mechanical behavior under uniaxial load in bending tests cannot be measured directly; instead, parameters can only be identified through measurement of the bending moment. The stress and strain distribution in the thickness direction of the specimen is non-uniform, and the same bending moment may correspond to different stress distributions. Therefore, parameter identification using bending moment may lead to inaccurate results [23].

The in-plane shear test is another common method to measure the hardening behavior of sheet metal. This test can effectively measure large strain deformation while avoiding plastic instability, and can be easily carried out on the mechanical tensile testing machine with special fixtures [24]. The American Society for Testing and Materials standard ASTM B831 [25] provides a simple shear test with a single-shear-zone specimen to investigate the mechanical properties of thin sheets of aluminum alloy under shearing. However, this specimen cannot be used to test the mechanical properties under cyclic shear loading. Merklein and Biasutti [26] developed a modified simple shear specimen by rotating the opening in the shear zone based on this standard; this modification allowed for the application of cyclic loading. Luo et al. [27] investigated the hardening and shear failure of AA6061-T6 sheets using a newly designed simple shear specimen. Although the shape of this kind of shear specimen with a single shear zone is simple and easy to process, it is also easy to rotate during the test, resulting in undesired deformation and premature failure of the specimen. K.Miyauchi [28] firstly proposed a simple shear test using a specimen with two rectangular shear zones. The specimen has a symmetrical structure, and the torque generated in the shear zone during the shear process can be balanced, so stable cyclic loading can be achieved. However, Pham [29] pointed out that the lack of constraints at the end of the rectangular shear zone may result in uneven local strain, which can lead to an end effect. This end effect has an impact on the accuracy of the test results. G ‘Sell et al. [30] proposed that a long and thin shear zone can be used to reduce the influence of the end effect on stress distribution. However, there is another issue with the rectangular shear zone: the diagonal of the rectangular shear zone easily shows crack under large-strain cyclic loading [31].

This paper proposes a V-shaped double-shear-zone specimen, which aims to achieve stable and uniform pure shear test conditions under large-strain cyclic loading. Initially, a V-shaped double-shear-zone specimen was designed to achieve uniform and stable pure shear deformation under large-strain cyclic loading. The feasibility of the V-shaped double-shear-zone simple shear test was verified by analyzing the stress and strain of the shear zone during experiments conducted under different cyclic loading conditions. Furthermore, the mechanical properties of Q890 high-strength steel sheets under different cyclic loading conditions were tested by a V-shaped double-shear-zone simple shear test. The study revealed the impact of shear pre-strain and loading cycles on the Bauschinger effect of Q890 high-strength steels.

## 2. Double-Shear-Zone Specimen for Cyclic Loading Experiments

### 2.1. Shape of Shear Specimen

The Miyauchi-type shear specimen can be used to study the mechanical properties of sheet metal under cyclic loading because of its symmetrical double shear zone, which can balance the torsion generated during the shear process [32]. However, stress concentration occurs at the diagonal position of the rectangular shear zone, and the shear deformation expands to the clamping regions, causing undesired boundary effects and non-uniform deformation of the shear zone, resulting in a deviation between the test results and the ideal pure shear state [33]. Additionally, under large shear strain, since the specimen is subjected to a large clamping force from the fixture, the two diagonals of the rectangular shear region are easily cracked during the cyclic loading process, which means it cannot be continued [34] and the shear strain limit of the material cannot be obtained.

**Figure 1:**
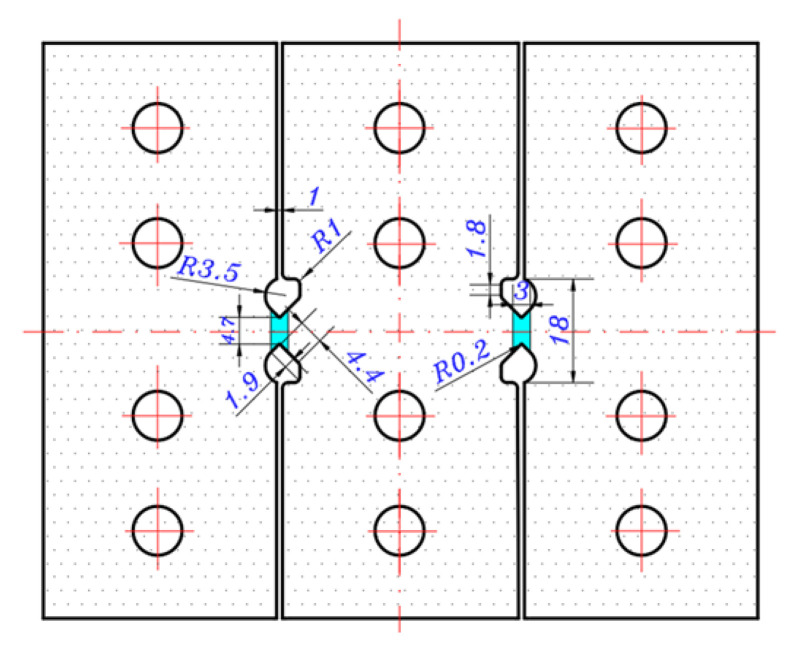
Schematic diagram of the dimensions of a simple shear specimen with the V-shaped double shear zone.

Therefore, this paper optimizes the Miyauchi-type shear specimen and proposes a V-shaped double-shear-zone specimen, the shape and key dimensions of which are shown in Figure 1. The new simple shear specimen consists of two V-shaped open shear zones and three clamping regions, which show mirror symmetry along the width and length directions. This design ensures that the torque generated by the shear zones on both sides is balanced and thus avoids torsional deformation during the shear process. The size of the shear zone is intentionally designed to be small while its adjacent area is large, the end of the shear zone is a V-shaped opening with a rounded corner tip and the spacing between the shear zone and the clamping regions is narrow. These designs can concentrate the shear force in the shear zone, reduce the stress concentration near the edge of shear zone and thus avoid local non-uniformity of the strain. As a result, the boundary effect between the shear zone and the clamping regions is significantly reduced, and nearly uniform pure shear deformation is developed in the shear zone. Besides that, twelve holes are symmetrically designed on the specimen to ensure that it can be firmly fixed to the fixture with bolts.

**Figure 2:**
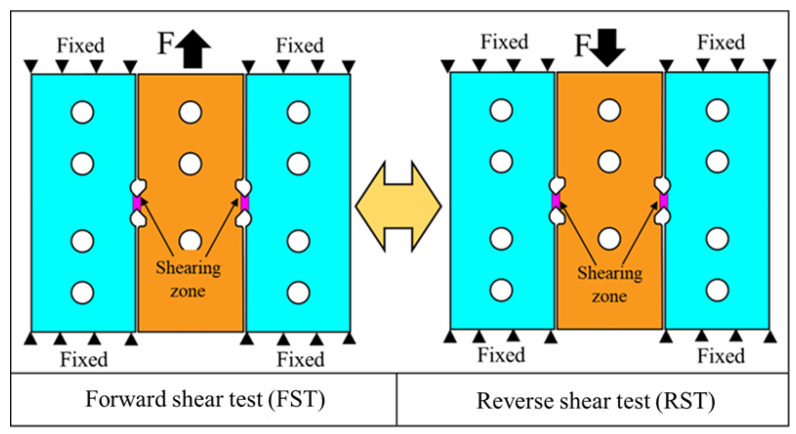
Schematic diagram of forward- and reverse-shear tests.

The principle of using this double-shear-zone specimen for cyclic loading experiments is illustrated in Figure 2. The clamping regions on both sides of the specimen are fixed, and forward- or reverse-shear deformation of the shear zone can be achieved by applying tension or pressure to the middle clamping region. This procedure can be termed forward-shear test (FST) and reverse-shear test (RST). Cyclic loading experiments can be conducted by continuously varying the tension and compression loading.

### 2.2. Shear Deformation Characteristics of New Specimens from FE Analysis

**Figure 3:**
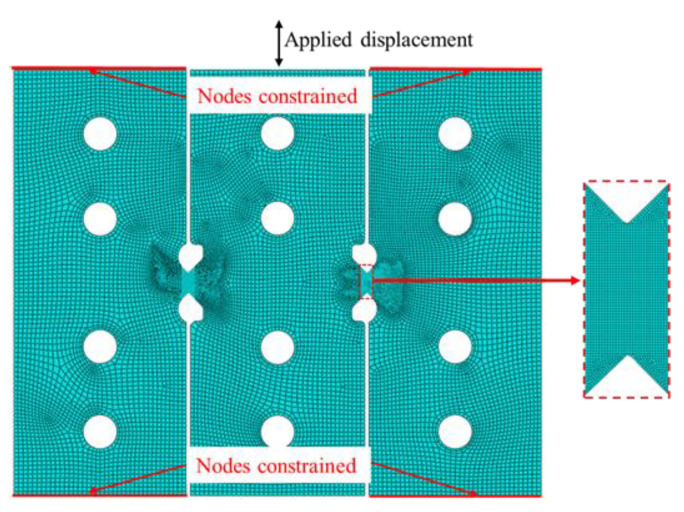
Finite element model for shear test.

In order to examine the deformation characteristics of the new shear specimen, including the pure shear state and the uniformity of stress–strain distribution, finite element simulations of shear experiments were performed using the ABAQUS6.14. The simulation model of the simple shear specimen with a V-shaped double shear zone (excluding clamping part) is shown in Figure 3. The four-node reduced integration shell elements (S4R) were used in the simulation, with the adoption of hourglass control. Five integration points were set in the thickness direction. Based on analyzing the impact of grid size on the uniformity of shear deformation, 1.0 mm × 1.0 mm was chosen as the in-plane mesh size of the clamping regions and 0.1 mm × 0.1 mm for the two shear zones. The two ends of the left and right clamping regions of the specimen were set as fixed constraints, and the axial velocity of 0.5 mm/min was set at one end of the middle clamping region of the specimen. The initial thickness of the specimen was 2.0 mm. The specimen is defined as an elastic–plastic body. The von Mises yield function was used as a material model in the FEA. The mechanical property parameters used in the simulation were obtained from a uniaxial tension test (UT).

The stress triaxiality *η_T_* and the Lode coefficient *μ_σ_* can reflect the stress and strain states. For isotropic materials, *η_T_* and *μ_σ_* should be 0 when they are in a pure shear stress state [35]. Therefore, *η_T_* and *μ_σ_* are used to evaluate the shear stress state of the new specimen. The expressions of *η_T_* and *μ_σ_* are as follows:

(1) $$\eta_{T}=\frac{1}{3}\left(\sigma_{1}+\sigma_{2}+\sigma_{3}\right)/\sqrt{\frac{1}{2}\left({\left(\sigma_{1}-\sigma_{2}\right)}^{2}+{\left(\sigma_{1}-\sigma_{3}\right)}^{2}+{\left(\sigma_{2}-\sigma_{3}\right)}^{2}\right)}$$

(2) $$\mu_{\sigma }=\left(2\sigma_{2}-\sigma_{1}-\sigma_{3}\right)/\left(\sigma_{1}-\sigma_{3}\right)$$

**Figure 4:**
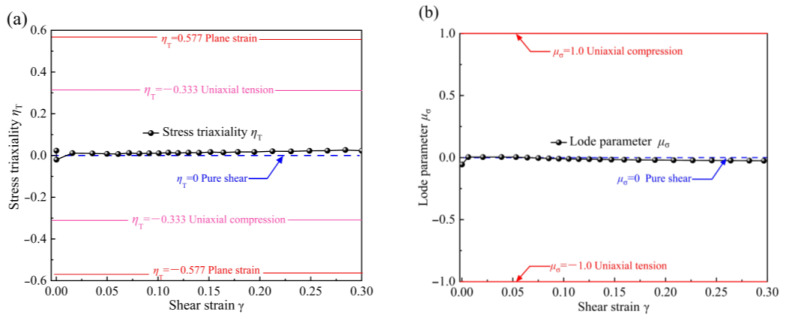
Evolution of stress triaxiality and Lode coefficient with increasing shear strain: (**a**) stress triaxiality *η_T_*; (**b**) Lode coefficient *μ_σ_*.

Figure 4 shows the evolution of *η_T_* and *μ_σ_* with the shear strain *γ* at the middle position of the shear zone. The stress triaxiality *η_T_* reaches its maximum value, around 0.023, during the initial elastic deformation stage. It then gradually decreases with increasing shear strain, eventually approaching 0. Correspondingly, the Lode coefficient *μ_σ_* is about 0.056 in the initial elastic deformation stage, and then increases gradually with increasing shear strain and approaches 0. During the plastic deformation process, both *η_T_* and *μ_σ_* are close to 0, indicating that the V-shaped double-shear-zone specimen experiences almost a pure shear stress state during deformation.

**Figure 5:**
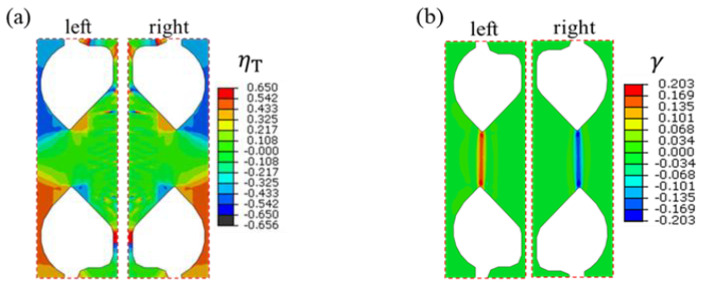
Distributions of stress triaxiality and shear strain of shear specimen with mean shear strain of 0.203: (**a**) stress triaxiality *η_T_*; (**b**) shear strain *γ*.

Figure 5 shows the distributions of *η_T_* and *γ* within both the left and right shear zones at a shear strain of 0.203. The results reveal that the distribution of *η_T_* in the whole shear zone is uniform and close to 0. The areas where *η_T_* deviates from 0 are away from the shear zone, indicating that the stress generated by the equilibrium torsion has been weakened towards the end of the shear zone, ultimately having little impact on the stress distribution within the zone. The shear strain is uniformly concentrated in the shear zone, with small edge effects. Consequently, the aforementioned results demonstrate that the proposed specimen can achieve a uniform and stable pure shear deformation in the in-plane shear experiment.

Through the analysis performed, the new simple shear specimen with a V-shaped double shear zone designed in this paper has the following characteristics:

(1) The V-shaped opening in the shear zone can effectively reduce the end effect and boundary effect, thereby achieving approximately uniform pure shear deformation.
(2) The symmetric structure of the shear specimen can effectively avoid torsional deformation and easily achieve stable forward/reverse pure shear deformation, even under large strains. It is particularly suitable for studying the mechanical properties of sheet metal under cyclic loading conditions.

## 3. Material and Experiments

### 3.1. Mechanical Properties of Q890 High-Strength Steel

Q890 high-strength steel has been widely used in the field of mechanical manufacturing as it has high strength and is economical [36]. Compared with traditional low-carbon steel, such as Q460 and Q690, the ductility of Q890 high-strength steel is lower but the yield strength is higher, and there are few reports on its mechanical properties under cyclic loading. Therefore, here, the mechanical properties of Q890 high-strength steel sheets under cyclic loading are studied using the proposed specimen.

**Figure 6:**
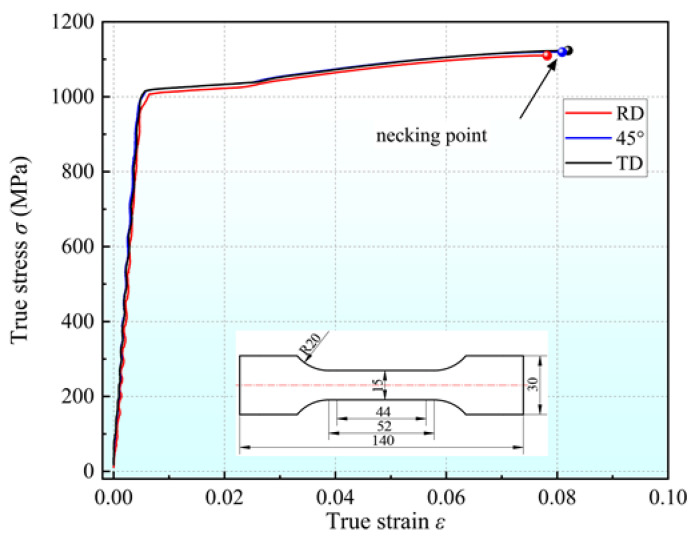
Flow stress–strain curves for Q890 in different orientations.

All specimens used for this paper were cut from 2 mm thick cold-rolled steel sheets, the chemical composition of which is shown in Table 1. Uniaxial tensile specimens were designed according to the Chinese Standard GBT 228.1-2010 [37], with a gauge length of 44 mm and a total length of 140 mm. Three uniaxial tensile tests were carried out at 0° (RD), 45° and 90° (TD) relative to the rolling direction. The tensile tests were performed using an electronic universal testing machine. The loading speed was 1.0 mm/min, and the approximate engineering strain rate was 0.0005 s^−1^. The strain field was measured by a three-dimensional digital image system (DIC) during the test. The test in each orientation was repeated three times and the results were found to be very repeatable. For this reason, only one result per orientation is plotted in Figure 6. It can be seen that the elongations, initial yield stress and hardening rates in the three orientations are very similar. The elongations in three orientations are all around 15%. The difference between the yield and tensile strengths is very small. The yield strength is about 1020 MPa and the tensile strength is about 1106 MPa, and the yield-to-tensile strength ratio is approximately 0.92 as shown in Table 2. In addition, the anisotropic coefficients are all close to 1 in three orientations, so the material can be considered to be in-plane-isotropic.

### 3.2. Experimental Setup

**Figure 7:**
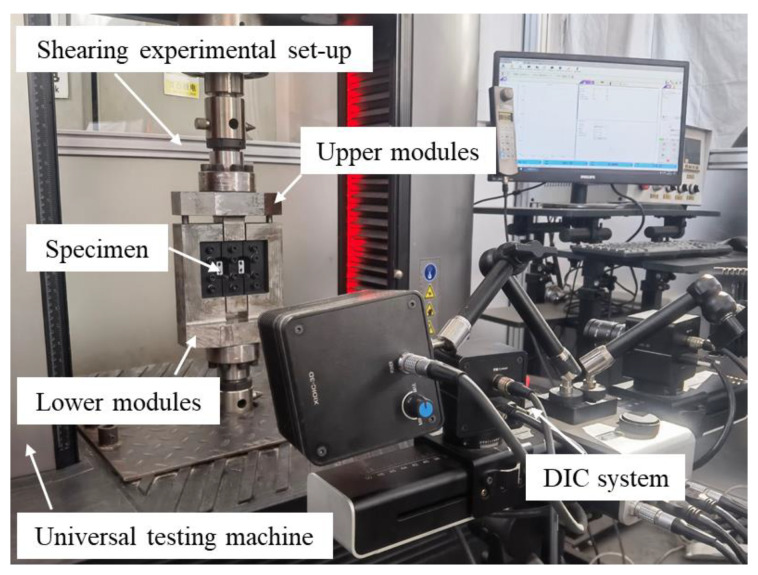
Experimental setup and strain measurement system.

The shearing experimental setup of the V-shaped double-shear-zone specimen is shown in Figure 7. The upper and lower modules of the experimental setup were installed on the LE5105 100 KN electronic universal tensile testing machine, and the lower module was completely fixed. The clamping regions on both sides of the shear specimen were completely fixed in the lower module through a clamp plate, which limited its torsion and end displacement. The middle clamping region of the shear specimen was installed in the upper module and was moved up and down by applying a load to the upper module, thus achieving forward- or reverse-shear experiments. The displacement range for the upper module was −15 mm to 15 mm. Specimens of thicknesses smaller than 10 mm could be tested by adjusting bolts and adding metal gaskets. The shear stress in the shear region was balanced and can be calculated by

(3) $$\tau =\frac{F}{2lt}$$

where *F* is the force applied to the shear specimen by the tensile machine, and *l* and *t* are the length and thickness of the shear zone of the specimen, respectively.

An open groove was designed between the clamping plates to expose the shear zone. Therefore, the deformation of the shear zone could be measured in real time by a DIC system. The DIC system has two cameras with a focal length of 50 mm, each with a full width of 2580 × 1936 pixels. The shear strain γ is calculated as follows:

(4) $$\gamma =\tan\left(\theta \right)\;$$

where *θ* is the shear angle, measured by the DIC system.

### 3.3. Experimental Program

**Figure 8:**
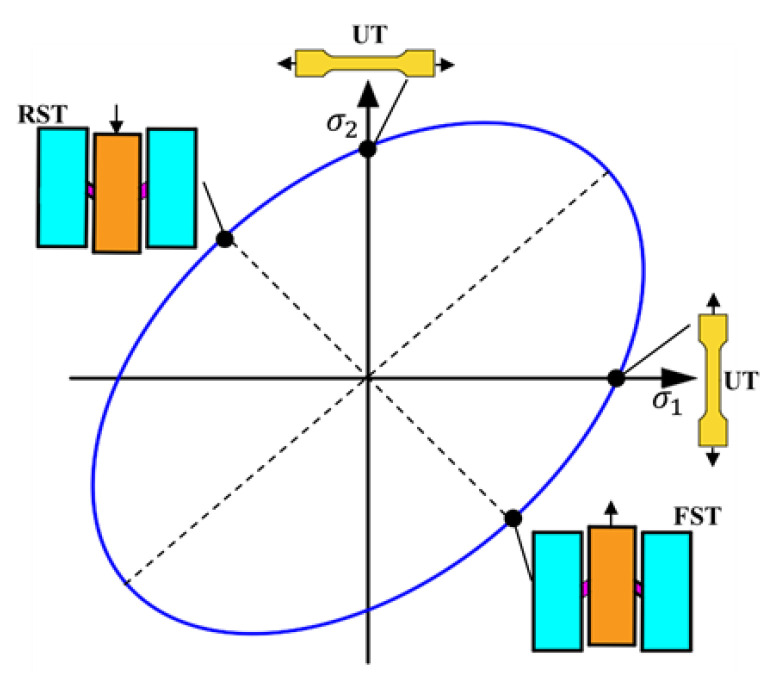
Schematic of the stress states corresponding to uniaxial tensile test and simple shear experiment on the yield surface in the (*σ*_1_ − *σ*_2_) space.

Firstly, simple shear experiments were carried out to analyze the uniformity of deformation in the shear zone. The results were then compared with those of the uniaxial tensile test to verify the ability of the new shear specimen to obtain large strains. Figure 8 shows the yield surface of the uniaxial tensile test and the simple shear test in a particular (*σ*_1_ − *σ*_2_) stress space.

Furthermore, different cyclic shear experiments were conducted on Q890 high-strength steel using the V-shaped double-shear-zone specimen. The goal was to analyze the impact of pre-strain and cyclic loading frequency on the Bauschinger effect of Q890 high-strength steel sheets. Three types of cyclic shear experiments were included as follows:

(1) Single-cycle shear experiment

Single-cycle shear experiments with shear pre-strains of 0.01~0.10 and 0.15 were conducted to study the impact of pre-strain on Bauschinger effect. The experimental process was as follows: the shear specimen was loaded forward to reach the pre-strain and then loaded in the reverse direction until the specimen fractured.

(2) Multi-cycle shear experiments with fixed pre-strain

The Bauschinger effect of Q890 high-strength steel under multiple cyclic loading was studied through four cycle shear experiments under shear pre-strains of 0.04 and 0.10. The experimental process was as follows: firstly, the shear specimen was forward-sheared to the pre-strain, and then reverse-sheared to the strain $-\gamma_{0}$, cycled four times, and the specimen was loaded until fracture during the fourth reverse shearing.

(3) Multi-cycle shear experiments with gradually increasing pre-strain

Multi-cycle shear experiments with gradually increasing pre-strain were conducted, the initial pre-strain $\gamma_{0}$ and strain increments Δγ were both 0.02. The experimental process was as follows: For the first loading cycle, the shear specimen was forward-sheared to $\gamma_{0}$, and then reverse-sheared to the same shear strain $-\gamma_{0}$. In the second loading cycle, the shear pre-strain was increased Δγ, that is, the second forward and reverse shearing until the strain reached $\gamma_{0}+\Delta \gamma$ and $-\gamma_{0}-\Delta \gamma$, respectively. This was cycled five times, and the fifth forward shearing was carried out until the strain reached 0.1, and then reverse loading was performed until the specimen fractured.

It should be noted that to maintain consistency with the strain rate used in the uniaxial tensile test, the loading speed of all shear experiments was set to 0.5 mm/min, resulting in a corresponding shear strain rate of approximately 0.0003 s^−1^. In addition, all shear specimens were cut along the rolling direction (RD).

### 3.4. Bauschinger Stress Parameter

**Figure 9:**
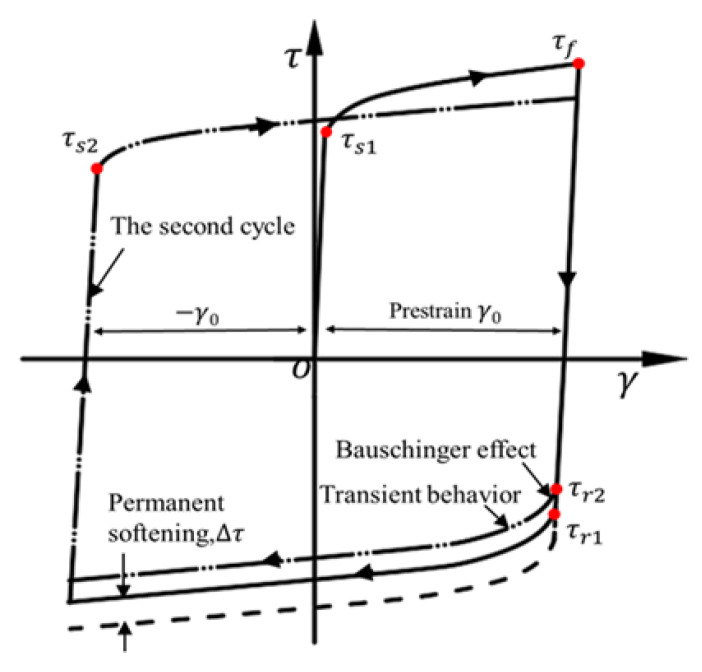
Schematic of the cyclic loading curve to illustrate the Bauschinger effect, transient behavior and permanent softening. (The dashed lines are the forward loading curves rotated by 180°).

The Bauschinger effect was first reported by J. Bauschinger [7] in the mechanical properties of metal materials in 1886. It indicates that the plastic strain hardening caused by forward loading during metal plastic processing leads to plastic strain softening (yield strength reduction) of metal materials during subsequent reverse loading, as shown in Figure 9. The Bauschinger effect can be quantified in various forms [38,39,40]. Bauschinger stress parameter (BSP) is often used to study the relationship between Bauschinger effect and pre-strain [41]. In this paper, the Bauschinger effect of Q890 high-strength steel sheet under different cyclic loading conditions is evaluated by BSP. The BSP is defined as

(5) $$BSP=\frac{\left|\tau_{f}\right|-\left|\tau_{r}\right|}{\left|\tau_{f}\right|}$$

where $\tau_{f}$ represents the flow stress from forward shear to pre-strain, and $\tau_{r}$ represents the initial yield stress when reverse shearing is performed after forward shear, as shown in Figure 9.

## 4. Results and Discussion

### 4.1. Simple Shear Experiment (SSE)

Three simple shear experiments were conducted, and the results were in good agreement. Hence, only one result was analyzed. According to the results of the uniaxial tensile tests, it can be approximated that the Q890 material is isotropic in-plane. Therefore, the simple shear stress–strain curve can be converted into an equivalent stress–strain curve by the principle of equivalent plastic work according to the von Mises yield criterion [42]. The conversion relationship is

(6) $$\left\{\begin{array}{l} \sigma_{eq}=\sqrt{3}\tau \\ \varepsilon_{eq}=\frac{1}{\sqrt{3}}\gamma \end{array}\right.$$

where *σ_eq_* and *ε_eq_* represent the equivalent stress and equivalent strain, respectively, and *τ* and *γ* represent the shear stress and shear strain, respectively.

**Figure 10:**
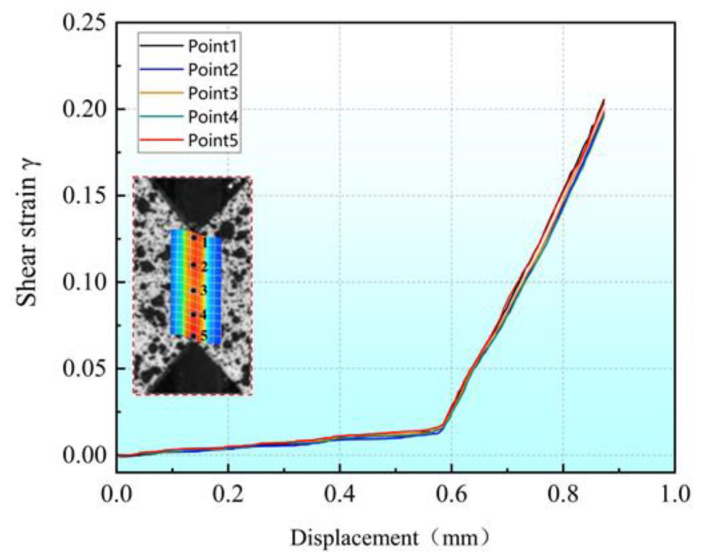
Evolution of shear strains at five different positions in the shear zone with the displacement of the middle plate.

Five sample points in the shear zone of a simple shear experiment were selected, and the relationships between the shear strain of each point and the displacement of the middle module are shown in Figure 10. It can be seen that the shear strain of each point is similar, and the maximum difference is only 3.7% when the maximum displacement is reached. This suggests a relatively even distribution of shear strain within the shear region with little end effect. For comparison purposes, all shear experiments were used to calculate the shear strain from point 3, located at the center of the shear zone.

**Figure 11:**
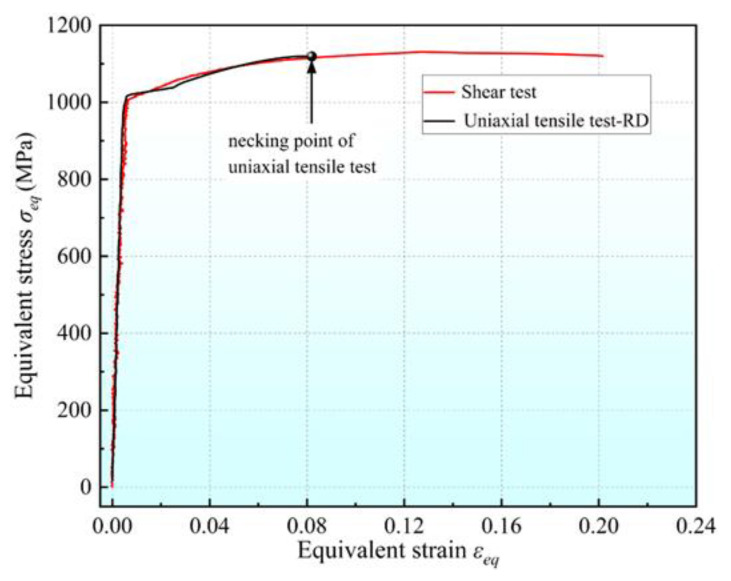
Equivalent stress–strain curves from uniaxial tensile and simple shear tests.

The equivalent stress–strain curve obtained by the simple shear experiment and the flow stress–strain curve in the RD direction obtained by the UT are shown in Figure 11. This plot establishes that the two curves basically coincide before the necking of the uniaxial tensile specimen, which verifies the in-plane isotropy of the material. The yield characteristics of Q890 can be well described by the von Mises criterion. Q890 exhibits a uniform strain of 0.082 in the UT, but that test is of limited usefulness for acquiring the large strain hardening curve, while the maximum equivalent plastic strain generated in the SSE is about 0.23, which is much larger than the result of the UT. Therefore, the simple shear experiment using the proposed shear specimen in this paper is more advantageous for studying the mechanical properties of sheet metal under large strain.

### 4.2. Single-Cycle Shear Experiment

**Figure 12:**
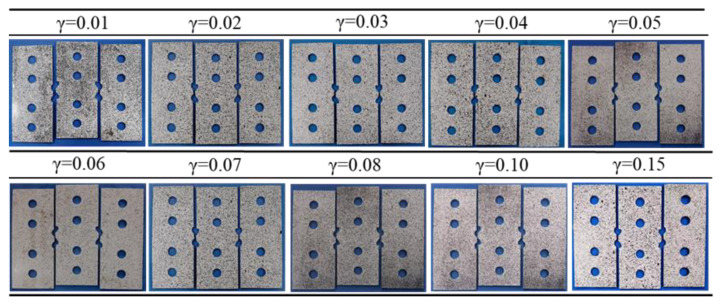
Shear specimens after single-cycle shear experiments.

**Figure 13:**
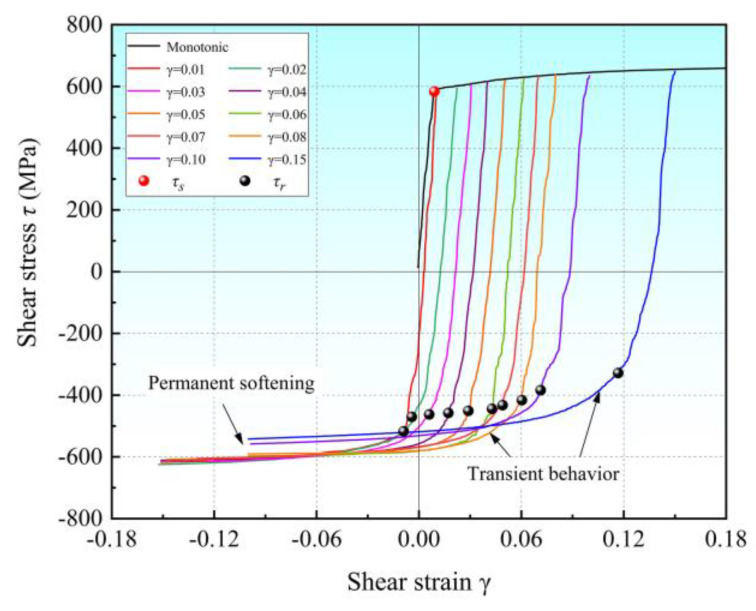
Flow stress–strain curves of single-cycle shear experiments with different shear pre-strains.

The specimens after the single-cycle shear experiments are shown in Figure 12. The data obtained from the experiments are shown in Table 3. The shear stress–strain curves obtained from the experiments are plotted in Figure 13, and the initial yielding stresses of forward and reverse loading are marked in the figure. The results show that the single-cycle shear experiments with different pre-strains were successfully conducted by using the proposed shear specimen. Q890 high-strength steel exhibits a significant Bauschinger effect, and with the increase in the forward-shear pre-strain, the reduction in the initial yield stress in the subsequent reverse shear of Q890 high-strength steel increases. When the forward-shear pre-strain increases from 0.01 to 0.15, the reduction in the reverse-shear initial yielding stress *τ_r_* increases from 58.4 MPa to 241.0 MPa compared to the forward-shear yield strength *τ_r_*. Moreover, transient softening characterized by hardening rate stagnation occurs during reverse loading. With the increase in shear pre-strain, the transient softening phenomenon is more obvious. In addition, the softening amount gradually increases with the increase in shear pre-strain, indicating that the permanent softening of Q890 gradually increases with the increase in shear pre-strain.

**Figure 14:**
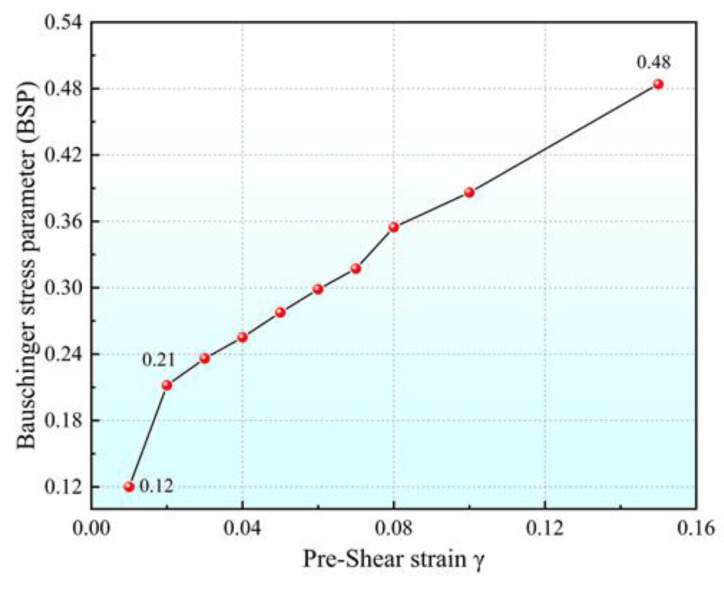
Variation in BSP with shear pre-strain.

Figure 14 shows the variation in BSP with shear pre-strain obtained through single-cycle shear experiments. BSP increases with the increase in shear pre-strain. When the shear pre-strain increases from 0.01 to 0.15, BSP increases from 0.12 to 0.48. When the shear pre-strain increases from 0.01 to 0.02, the BSP growth rate is twice as fast. This shows that the Bauschinger effect of Q890 high-strength steel increases with the increase in shear pre-strain, and increases faster at lower shear pre-strains.

### 4.3. Multi-Cycle Shear Experiments with Fixed Pre-Strain

**Figure 15:**
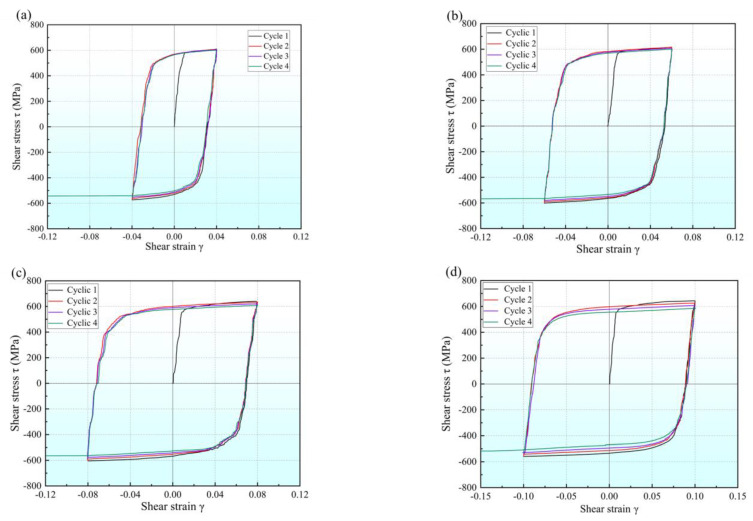
Flow stress–strain curves of multi-cycle shear experiments with fixed shear strain: (**a**) *γ* = 0.04; (**b**) *γ* = 0.06; (**c**) *γ* = 0.08; (**d**) *γ* = 0.10.

The data obtained from four cycle shear experiments with pre-strains of 0.04 and 0.10, respectively, are shown in Table 4, and the shear stress–strain data are plotted in Figure 15. The results show that the proposed shear specimen can be successfully used in multiple-cyclic shear tests, and is very stable even under large strain. For Q890 high-strength steel, in the multi-cycle forward- and reverse-shear experiments with fixed pre-strain, the forward-shear yield stress $\tau_{s}^{i}$ (the superscript *i* indicates the number of cycle, here *i* ≥ 2) is lower than the initial shear yield stress $\tau_{s}^{}$, but significantly greater than the reverse-shear yield stress $\tau_{r}^{i-1}$ (*i* ≥ 2) of the previous cycle. With the increase in loading cycles, both the forward- and reverse-shear yield stresses gradually decrease, and the greater the pre-strain, the greater the decrease. At the fourth cycle, when the cyclic shear pre-strain is increased from 0.04 to 0.10, the forward-shear yield stress is 499.7 MPa and 443.5 MPa, respectively. This represents yield strength reduction rates of 13.7% and 23.3%, compared with the original material. Similarly, the reverse-shear yield strength decreases to 424.4 MPa and 373.6 MPa, respectively, with a yield strength reduction rate of 26.7% and 35.4%, respectively, compared with the original material. Both the flow stress corresponding to the forward and reverse loading at the maximum shear strain gradually decreases with the increase in loading cycles, indicating that the permanent softening increases with the increase in loading cycles.

**Figure 16:**
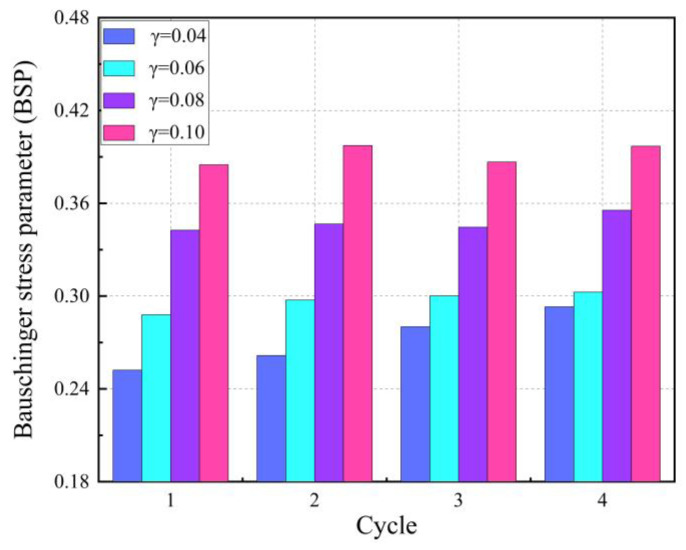
The relationship between BSP and loading cycles in multiple-cyclic shear experiments with fixed pre-strain.

Figure 16 shows the relationship between BSP and loading cycles under fixed pre-strain. It can be observed that BSP varies greatly with the increase in pre-strain, but the change is small with the increase in loading cycles. This implies that although the yield strength of Q890 high-strength steel decreases with the increase in loading cycles, the back stress caused by dislocation in the material does not change significantly with the loading cycles.

### 4.4. Multi-Cycle Shear Experiments with Gradually Increasing Pre-Strain

**Figure 17:**
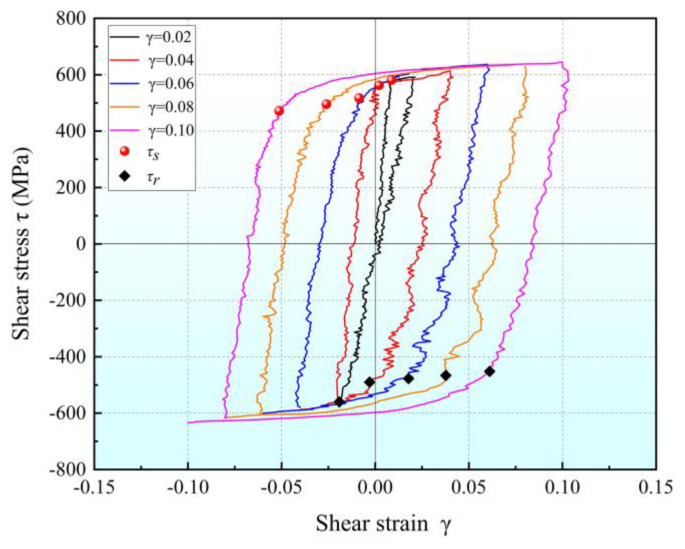
The shear stress–strain curves for cyclic shear tests with increasing pre-strain.

The flow stress–strain curves obtained from multi-cycle shear experiments with gradually increasing pre-strain are plotted in Figure 17, and both the forward- and reverse-shear yield stresses are marked in the figure. The results show that when the loading cycle increases and the shear pre-strain increases by 0.02, both the forward- and reverse-shear yield stresses are significantly reduced. When the shear pre-strain increases from 0.02 to 0.1, the forward-shear yield stress decreases from 578.2 MPa to 471.8 MPa, while the reverse-shear yield stress decreases from 508.7 MPa to 444.8 MPa.

**Figure 18:**
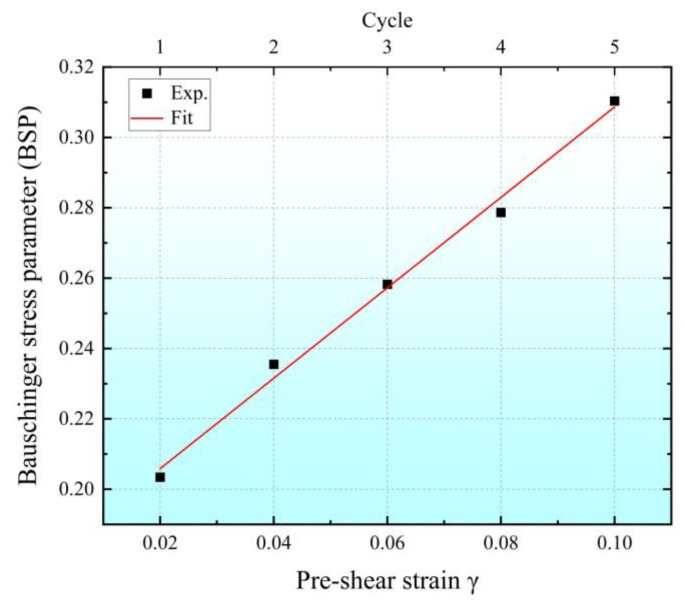
Relationship between BSP and pre-strain and loading cycles.

Figure 18 shows the relationship between BSP and shear pre-strain in multi-cycle shear experiments with gradually increasing pre-strain. Each experimental point in the diagram is the BSP obtained for each loading cycle. The BSP is affected by both the shear pre-strain and the loading cycle. Based on the above analysis, it can be seen that the shear pre-strain has a greater impact on the BSP, and the Bauschinger effect becomes more significant with the increase in shear pre-strain and loading cycles.

## 5. Conclusions

In this paper, a V-shaped double-shear-zone specimen is proposed, and its shear deformation characteristics are analyzed by FEM. Using this proposed specimen, simple shear experiments and three different cycle shear experiments including single-cycle shear experiment and multi-cycle shear experiments with both fixed pre-strain and gradually increasing pre-strain, respectively, are conducted to analyze the uniformity of deformation in the shear zone and the effect of pre-strain and the number of cyclic loads on the Bauschinger effect of Q890 high-strength steel sheet. The main conclusions are as follows:

1. The V-shaped double-shear-zone specimen can largely eliminate the end effect and the boundary effect, so that uniform pure shear deformation can be achieved in the shear zone.
2. A simple shear test with the proposed specimen can effectively avoid torsional deformation, and the resulting strains are significantly higher than the uniform elongation in the UT. Moreover, it is easy to achieve stable forward/reverse cyclic loading experiments, and even under large strains.
3. Q890 high-strength steel exhibits a significant Bauschinger effect, which is more pronounced with the increase in shear pre-strain and loading cycles. However, relatively speaking, the influence of shear pre-strain on the Bauschinger effect is more significant, and the smaller the pre-strain, the more significant it is.
4. For Q890 high-strength steel, in both multi-cycle shear experiments with fixed pre-strain and gradually increasing pre-strain, respectively, both the forward- and reverse-shear yield stresses gradually decrease as the loading cycles increase, and the greater the pre-strain, the greater the decrease; the forward-shear yield stress $\tau_{s}^{i}$ (*i* ≥ 2) is lower than the initial shear yield stress *τ_s_*, but significantly greater than the reverse-shear yield stress of the previous cycle $\tau_{r}^{i-1}$ (*i* ≥ 2).
5. The permanent softening of Q890 gradually increases with an increase in either shear pre-strain or loading cycles.
6. The wall thickness of the specimen can be a limiting factor for the conclusion of this study. When the wall thickness of the specimen is too thick, the accuracy of the results may be affected.

## 6. Future Study

In future studies, the following aspects could be investigated, such as the effect of specimen wall thickness on shear deformation, the establishment of intrinsic structure relations applicable to cyclic loading and the effect of pre-strain on the forming limit.

## Figures and Tables

**Table 1:** Chemical composition of Q890 steel.

Chemical Composition	C	Si	Mn	P	S	Cu	Cr	Ni	Mo	B	V	Nb	Ti	CEV
**content/%**	0.2	0.8	2.0	0.025	0.015	0.5	1.5	2.0	0.7	0.005	0.12	0.06	0.05	0.72

**Table 2:** Mechanical properties of Q890 steel.

Direction	Young’s Modulus/GPa	Yield Stress/MPa	Tensile Strength/MPa	Elongation/%	*r*-Value
0°	204.5	1020	1115	14.3	0.97
45°	203.9	1015	1106	14.6	0.98
90°	205.6	1026	1102	14.1	1.01

**Table 3:** Single-cycle shear experiment data under different pre-strains.

Shear Pre-Strain	*τ_s_*/MPa	*τ_f_*/MPa	*τ_r_*/MPa	|*τ_s_*| − |*τ_r_*|/MPa
0.01	578.2	589.5	−519.8	58.4
0.02	578.9	602.6	−474.9	103.9
0.03	577.6	608.1	−464.5	113.1
0.04	579.1	615.3	−458.3	120.8
0.05	586.0	624.3	−451.0	135.0
0.06	578.1	630.2	−442.1	136.0
0.07	577.9	636.1	−434.4	143.6
0.08	578.0	638.4	−412.7	165.3
0.10	578.2	645.6	−396.3	181.9
0.15	579.3	654.6	−338.3	241.0

**Table 4:** Results of multi-cycle shear experiments with fixed pre-strain.

Shear Pre-Strain	Cyclics	*τ_s_*/MPa	*τ_f_*/MPa	*τ_r_*/MPa	|*τ_s_*| − |*τ_r_*|/MPa
0.04	1	579.1	612.8	−458.3	120.8
	2	511.6	607.6	−448.7	62.9
	3	502.8	604.4	−435.1	67.7
	4	499.7	600.3	−424.4	75.3
0.06	1	578.1	622.4	−443. 3	134.8
	2	485.8	618.6	−434.7	51.1
	3	483.5	609.1	−426.3	57.2
	4	477.5	601.4	−419.5	58.0
0.08	1	578.1	635.2	−417.6	160.5
	2	446.7	627.3	−409.7	37.0
	3	428.9	615.6	−403.5	25.4
	4	423.4	607.3	−391.4	32.0
0.10	1	578.2	644.1	−396.2	182.0
	2	447.5	627.7	−384.3	63.2
	3	453.7	607.1	−378.4	75.3
	4	443.5	599.6	−373.6	69.9

## Data Availability

All the data from the experiments, including videos and photos, are available upon request. Interested readers are encouraged to contact the corresponding author for the data.
